# Quantitative Studies on Structure-DPPH• Scavenging Activity Relationships of Food Phenolic Acids

**DOI:** 10.3390/molecules171112910

**Published:** 2012-11-01

**Authors:** Pu Jing, Shu-Juan Zhao, Wen-Jie Jian, Bing-Jun Qian, Ying Dong, Jie Pang

**Affiliations:** 1 Research Center for Food Safety and Nutrition, Key Lab of Urban Agriculture (South), Bor S. Luh Food Safety Research Center, School of Agriculture & Biology, Shanghai Jiao Tong University, Shanghai 200240, China; Email: bjqianfd@yahoo.com.cn (B.-J.Q.); 2 Department of Food Science and Engineering, School of Food and Biological Engineering, Jiangsu University, Zhenjiang 212013, China; Email: zshj_1012@126.com (S.-J.Z.); ydong@ujs.edu.cn (Y.D.); 3 College of Food Science, South China Agricultural University, Guangzhou 510642, China; Email: jianwenjie123@163.com; 4 College of Food Science, Fujian Agriculture and Forestry University, Fuzhou 350002, China; Email: pang3721941@163.com

**Keywords:** 3D-QSAR, phenolic acids, free radical scavenging, structure, CoMFA, CoMSIA

## Abstract

Phenolic acids are potent antioxidants, yet the quantitative structure-activity relationships of phenolic acids remain unclear. The purpose of this study was to establish 3D-QSAR models able to predict phenolic acids with high DPPH• scavenging activity and understand their structure-activity relationships. The model has been established by using a training set of compounds with cross-validated *q*^2^ = 0.638/0.855, non-cross-validated *r*^2^ = 0.984/0.986, standard error of estimate = 0.236/0.216, and F = 139.126/208.320 for the best CoMFA/CoMSIA models. The predictive ability of the models was validated with the correlation coefficient *r*^2^_pred_ = 0.971/0.996 (>0.6) for each model. Additionally, the contour map results suggested that structural characteristics of phenolics acids favorable for the high DPPH• scavenging activity might include: (1) bulky and/or electron-donating substituent groups on the phenol ring; (2) electron-donating groups at the *meta*-position and/or hydrophobic groups at the *meta-*/*ortho*-position; (3) hydrogen-bond donor/electron-donating groups at the *ortho*-position. The results have been confirmed based on structural analyses of phenolic acids and their DPPH• scavenging data from eight recent publications. The findings may provide deeper insight into the antioxidant mechanisms and provide useful information for selecting phenolic acids for free radical scavenging properties.

## 1. Introduction

Phenolic acids are a group of secondary plant metabolites, widely spread throughout the plant kingdom and in foods of plant origin [1]. Research on phenolic acids is carried out because of their biological and pharmacological properties, especially antioxidant activity [2].

The *ortho*-dihydroxyl substitution pattern is commonly regarded as important for the radical scavenging activities of phenolic acids [2,3,4]. Natella *et al*. reported that *para*-dihydroxyacids showed a higher radical scavenging activity than monohydroxyacids for both benzoic and cinnamic acid series using a competitive kinetics test [5]. However, Cai and co-workers found that a multiple-hydroxyl group pattern on the aromatic ring(s) appeared to be more important for hydroxybenzoic acids with high antioxidant activity than for hydroxycinnamic acids [4]. Actually, hydroxycinnamic acids showed higher antioxidant activities than the corresponding hydroxybenzoic acids because the carbon side chain structure in the phenol ring affected the DPPH• scavenging activity [6]. The additional hydroxyl group in the *para*-position of hydroxycinnamic acids with respect to the carbon substituent, compared with *meta-* or *ortho*-position, has been found to significantly increase radical scavenging activity [4]. The radical scavenging activity of phenolic acids with high activity was associated with methoxy groups no matter the substituent position [4]. Specifically, methoxy substituents on the ring enhanced the radical scavenging activity of *para*-hydroxyphenolic acids with regard to the COOH or carbon substitutent [5].

More recently quantitative structure activity relationship (QSAR) studies have served as an efficient tool to elucidate the structure-activity relationships of antioxidants, including chroman amides and nicotinyl amides [7], hydroxybenzalacetones [8], hydroxyflavonnes [9], phenolics [10] and wine polyphenols [11]. This study aimed to build the QSAR of the phenolic acid derivatives using the comparative molecular field analysis (CoMFA) and the comparative molecular similarity indices analysis (CoMSIA) methods to predict phenolic acids with the high DPPH• scavenging activity and also understand their quantitative structure-activity relationships. The study should provide information for antioxidant mechanism studies and for selecting phenolic acids with strong free radical scavenging properties.

## 2. Results and Discussion

### 2.1. CoMFA and CoMSIA Model

The statistic results for both CoMFA and CoMSIA models are shown in Table 1. The internal validation of leave-one-out cross-validated *q*^2^ and non-cross validated coefficient *r*^2^ are commonly applied as a criterion of robustness and predictive ability of a QSAR model. The commonly accepted values for a satisfactory QSAR model are *q*^2^ > 0.5 and *r*^2^ > 0.8 [12]. A highly predictive CoMFA model with LOO cross-validated *q*^2^ of 0.638 and correlation values *r*^2^ of 0.984 were obtained. The steric contribution and electrostatic contribution were 50.5% and 49.5% for the QSAR model. The standard error of estimate and F-test value were 0.236 and 139.126, respectively. The yielded *r*^2^_bs_ value 0.993 for CoMFA (SDbs = 0.007) further supported the statistical validity of the developed models.

**Table 1:** Statistical parameters of the CoMFA and CoMSIA models.

Statistics parameters	CoMFA model	CoMSIA model
*q* ^2^	0.638	0.855
*r* ^2^	0.984	0.986
*s*	0.236	0.216
*F*	139.126	208.320
PLS component	5	5
Field contribution		
Steric	0.505	0.058
Electrostatic	0.495	0.326
Hydrophobic		0.171
H-bond Donor		0.140
H-bond Acceptor		0.304
*r*^2^_bs_ (10 runs)	0.993	0.997
*SD*_bs_	0.007	0.003
*r* ^2^_pred_	0.971	0.996
*r_0_* ^2^	0.971	0.993
*k*	0.955	1.008
*(r* ^2^_pred_ *-r_0_* ^2^ *)/r* ^2^_pred_	0.000	0.003

*q*^2^: cross-validated correlation coefficient after the leave-one-out procedure; *r*^2^: non-cross-validated correlation coefficient; *s*: standard error of estimate; *F*: F-test value; PLS component: optimum number of components; *r*^2^_bs_: bootstrapping correlation; *SD*_bs_: bootstrapping standard deviation; *r*^2^_pred_: correlation coefficient for test set predictions; *r_0_*^2^: correlation coefficient for the regression through origin for experimental versus predicted activities; *k*: slope for regression through origin from experimental versus predicted.

The statistical results of the best CoMSIA model are also listed in Table 1. The good cross-validated *q*^2^ of 0.855 (>0.5) and the non-cross-validated coefficient *r*^2^ of 0.986 (>0.8) were obtained based on the steric, electrostatic, H-bond donor/acceptor, and hydrophobic fields that explained 5.8, 32.6, 14.0, 30.4 and 17.1% of the variance from the QSAR model, respectively. The non-cross-validated coefficient s and F value are 0.216 and 208.320, respectively. The cross-validation results suggested that CoMSIA model had a better predictive ability than the CoMFA model in this study. The yielded *r*^2^_bs_ value 0.997 for CoMSIA (SDbs = 0.003) further supports the statistical validity of the developed CoMSIA models. The predictive ability of the models was validated with the correlation coefficient *r*^2^_pred_ = 0.971/0.996 (>0.6) for each models, indicating that both CoMFA and CoMSIA models should have high predictive abilities for DPPH• scavenging activity of phenolic acids. The experimental and predicted activities in the training and test sets are shown in Table 2.

**Table 2:** Chemical structure and experimental activities of phenolic acid derivatives. 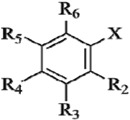

Compds		R_2_	R_3_	R_4_	R_5_	R_6_	X	Experimental pTEAC ^a^	Predicted pTEAC ^a^	
									CoMFA	CoMSIA
1 ^b^		H	OCH_3_	OH	H	H	COOH	2.127	2.252	2.497
2		H	OH	OH	H	H	COOH	0.309	0.568	0.657
3		H	H	OH	H	H	COOH	3.544	3.316	3.227
4		H	OCH_3_	OCH_3_	H	H	COOH	3.700	3.928	3.764
5		OOCH_3_	H	H	H	H	COOH	3.558	3.530	3.579
6		OCH_3_	H	H	H	H	COOH	3.667	3.595	3.913
7		H	OCH_3_	OH	OCH_3_	H	COOH	0.409	0.275	0.267
8		H	H	OCH_3_	H	H	COOH	3.608	3.464	3.475
9 ^b^		OH	H	H	OH	H	COOH	0.168	0.135	0.149
10		OH	H	H	H	H	COOH	3.535	3.624	3.572
11 ^c^		H	OH	OH	OH	H	COOH	0.179	0.104	0.265
12		H	OCH_3_	H	H	H	COOH	3.633	3.675	3.424
13 ^b^		H	H	OH	H	H	CH_2_COOH	3.482	3.297	3.602
14		OH	H	H	H	H	CH_2_COOH	3.501	3.484	3.575
15		H	OCH_3_	OH	H	H	CH_2_COOH	0.876	0.891	0.7809
16 ^b^		OH	H	OH	H	H	CH=CHCOOH	0.964	0.622	1.090
17		H	H	OH	H	H	CH=CHCOOH	3.349	3.457	3.479
18 ^b^		H	OH	OH	H	H	CH=CHCOOH	0.266	0.244	0.290
19		H	OH	OH	H	H	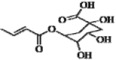	0.373	0.327	0.335
	20	H	H	H	H	H	CH=CHCOOH	3.594	3.468	3.483
	21 ^b^	H	OH	OH	H	H	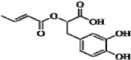	0.244	0.245	0.179
	22 ^b^	OCH_3_	H	H	H	H	CH=CHCOOH	3.561	3.184	3.258
	23	H	OCH_3_	OH	H	H	CH=CHCOOH	0.773	1.078	0.976
	24	H	OCH_3_	OH	OCH_3_	H	CH=CHCOOH	0.409	0.348	0.707

^a^ Experimental and predicted activities of the compounds which are expressed as pTEAC = −logTEAC; ^b^ compounds for test set; ^c^ compound for the template alignment.

### 2.2. External Validation of the CoMFA and CoMSIA Models

Although the internal validation with a high value of LOO cross-validated *q*^2^ obtained for both CoMFA and CoMSIA models was necessary and important, it was not sufficient for a QSAR model with a high predictive power [13]. Therefore, the predicting activity of an external test set with seven compounds was performed using all the CoMFA and CoMSIA models. The squared correlation coefficient values between the observed and predicted values of the test set compounds with intercept (*r*^2^) and without intercept (*r_0_*^2^) were calculated. The validation criteria proposed by Golbraikh and Tropsha applied to the regression analysis must satisfy the following conditions [12,13]: (1) *q*^2^ > 0.5; (2) *r*^2^_pred_ > 0.6; (3) [(*r*^2^_pred_-*r_0_*^2^)/*r*^2^] < 0.1; (4) 0.85 ≤ *k* ≤ 1.15, where *q*^2^ is the cross-validated correlation coefficient after the leave-one-out procedure; *r*^2^_pred_ is correlation coefficient for test set predictions; *r_0_*^2^ is the correlation coefficient for the regression through origin for experimental versus predicted activities. *k* is the slope of regression lines through the origin.The statistical results of the test set are given in Table 1. The values of the cross-validated *q*^2^ and correlation coefficient *r*^2^_pred_ for test set predictions, [(*r*^2^_pred_−*r_0_*^2^)/*r*^2^], and *k* of CoMFA and CoMSIA models satisfy the criteria.

### 2.3. CoMFA and CoMSIA Contour Maps Analysis

CoMFA and CoMSIA contour maps analyses were performed to visualize the important regions in 3D molecules where the steric, electrostatic, hydrogen-bond donor/acceptor, and hydrophobic fields may affect the DPPH• scavenging activity of the studied compounds. The weight of StDev*Coeff was used to calculate the field energies for all fields in CoMFA and CoMSIA models. The highly active compound **11** was shown as the template ligand for all contour map positions.

#### 2.3.1. CoMFA Contour Maps

**Figure 1:**
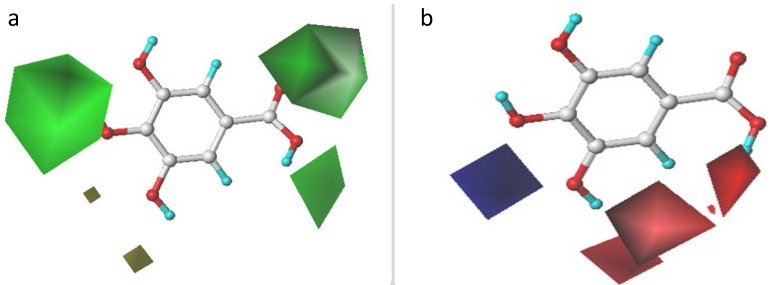
CoMFA contour maps. (**a**) steric contour map: the green is sterically favored for the activity , whereas the yellow is unfavorable; (**b**) electrostatic contour map: the blue contour for positive-charged substituent is favorable, whereas the red contour for the negative-charged substituent is favorable.

The steric contour map with sterically favorable (marked in green) and sterically unfavorable (marked in yellow) regions is shown in Figure 1a. A large green contour is located around the *para*-position of the phenol ring, indicating that the more bulky substituent is preferred to enhance the activity at this site. This was consistent with the experimental results, where a lower pTEAC value corresponds to a stronger DPPH• scavenging activity of a tested sample.

Compound **1** (R_3_: OCH_3_, R_4_: OH, pTEAC = 2.127) with a hydroxyl group on the *para*-position, had a higher activity than the corresponding compound **12** (R_3_: OCH_3_, R_4_: H, pTEAC = 3.633) without a bulky group at the same position. Similarly a bulky substituent at the *para*-position can also explain the activity differences between compound **17** (R_3_: H, R_4_: OH, pTEAC = 3.349) and compound **20** (R_3_: H, R_4_: H, pTEAC = 3.594). Another two green contours are located at the X-position (Figure 1a), suggesting that bulky substituents at the X-position appeared to be favorable for the DPPH• scavenging activity of phenolic acids. For example, compound **17** (X: CH=CHCOOH, pTEAC = 3.349), compound **14** (X: CH_2_COOH, pTEAC = 3.501) and compound **15** (X: CH_2_COOH, pTEAC = 0.876) showed higher activity than the corresponding compounds **3** (X: COOH, pTEAC = 3.544), **10** (X: COOH, pTEAC = 3.535), and **1** (X: COOH, pTEAC = 2.127), respectively. Therefore, the carbon side chain should follow a CH=CHCOOH > CH_2_COOH > COOH sequence for favorable DPPH• scavenging activity of the phenolic acids. That could be explainable by the steric factors that are thought to increase the activity by stabilizing the resultant phenoxy radical [8]. Additionally, the double bond in the side chain probably stabilized the radical by resonance [5].

However, it seems to difficultly explain the activities of all tested phenolic acids with the substituents in the *meta*- or *ortho*-position using the steric contour maps alone. Figure 1b shows the electrostatic contour map with electronegative favored (marked in red) and electropositive favored (marked in blue) regions. Two electronegative favored regions in Figure 1b were located at *meta*-positions (R_3_ and R_5_) and the regions between *ortho*- and X-positions, respectively, indicating that the presence of an electron-donating group or high electron density on these sites increased the activity. For example, compound **11** (R_3_: OH, R_5_: OH, pTEAC = 0.179) showed a greater activity than compound **2** (R_3_: OH, R_5_: H, pTEAC = 0.309), which had greater activity than compound **3** (R_3_: H, R_5_: H, pTEAC = 3.544) due to the number of electron-donating groups at the *meta*-position. Compound **15** (R_3_:OCH_3_, pTEAC = 0.876) had a higher activity than compound **13** (R_3_: H, pTEAC = 3.482). These observations were consistent with the electron delocalization and electron donation of an unshared pair of electrons from *o*-OCH_3_ in the *p*-orbital stabilizing the phenoxyl radical [10,14]. More electron-donating substituents contribute to facilitating phenoxyl radical formations, and *ortho*-hydroxyl group substituents should be effective in stabilizing the resultant phenoxyl radicals.

#### 2.3.2. CoMSIA Contour Maps

**Figure 2:**
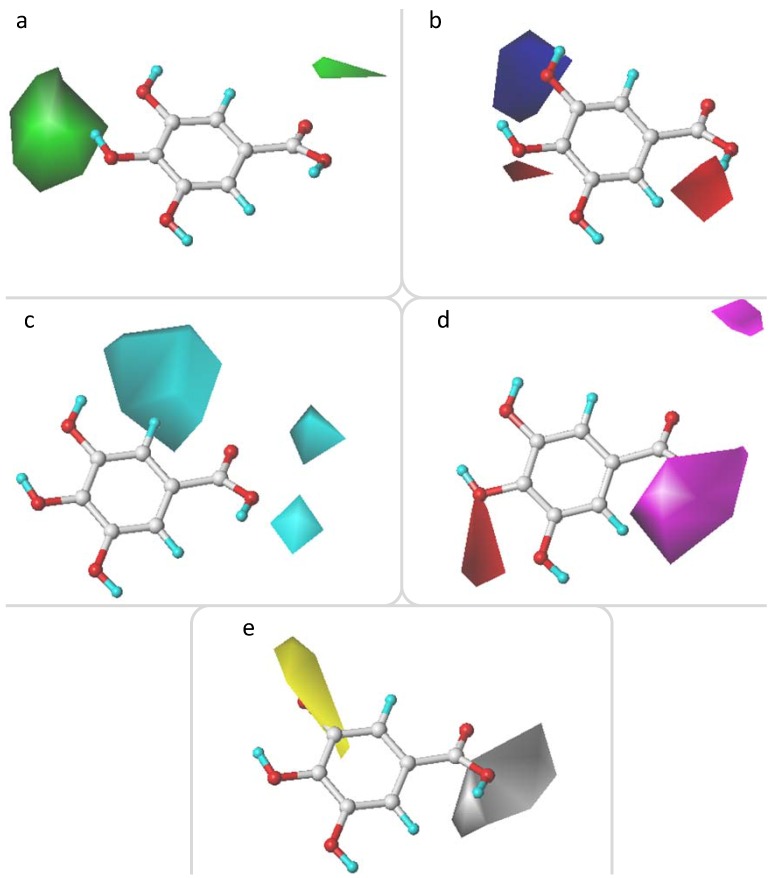
CoMSIA contour maps. (**a**) steric contour map: the green is sterically favored for the activity , whereas the yellow is unfavorable; (**b**) electrostatic contour map: the blue for positive-charged substituent is favorable, whereas the red for the negative-charged substituent is favorable; (**c**) hydrogen bond donor contour map: the cyan for hydrogen bond donors is favorable whereas the purple for hydrogen bond donors is unfavorable for the activity; (**d**) hydrogen bond acceptor contour map: the magenta for hydrogen bond acceptors is favorable for the activity whereas the red for hydrogen bond acceptors is unfavorable for the activity; (**e**) hydrophobic contour map: the yellow for hydrophobic group is favorable whereas the grey for hydrophobic group is unfavorable.

The contour maps for the CoMSIA model are shown in Figure 2. The steric contour map (Figure 2a) is similar to the CoMFA contour map (Figure 1a). For the electrostatic contour map, a small red region is located near to the *para*-position in Figure 2b, but does not exist in Figure 1b, suggesting that an electron-donating group or high electron density at the *para*-position should increase the activity of phenolic acid derivatives. For example, both compound **3** (R_4_: OH, pTEAC = 3.544) and **8** (R_4_: OCH_3_, pTEAC = 3.608) have bulky substituents at the *p*-position, but compound **3** showed higher activity than compound **8**, which is consistent with the presence of a higher electron density on the phenolic hydroxyl oxygen due to the electron-donating nature of the substituent making the compound more active [15].

The hydrogen-bond donor and hydrogen-bond acceptor fields in the CoMSIA model are shown in Figure 2c,d, respectively. The hydrogen-bond donor substituent around the cyan region (*ortho*- and X-position), and/or hydrogen bond acceptors around the magenta region (X-position) should be favorable for the DPPH• scavenging activity.

This could be explained by the fact that a hydrogen-bond donor in the phenol ring could be convenient for forming intermolecular hydrogen bonds and stabilizingfthe phenol radicals [3]. The hydroxyl or carboxyl substituent around the cyan region or magenta region should be favorable for high activity since they are both hydrogen-bond acceptors (hydroxyl or carbonyl substituents) and hydrogen-bond donors (hydroxyl substituents).

Figure 2e illustrates the CoMSIA hydrophobic contour, where hydrophobic groups in the yellow or grey regions are favorable or unfavorable for the DPPH• scavenging activity of phenolic acids, respectively. A small yellow region is located at the *meta*-position, suggesting that hydrophobic substituents in the region might enhance the activity of phenolic acids.

First, the phenolic acids could act either as hydrogen atom transferers or as electron transferers for their radical scavenging activity [16,17]. The developed CoMFA and CoMSIA models could explain structure/activity relationships of phenolic acids based on their DPPH• scavenging activity and some important conclusions have been drawn as follows: an electron-donating and/or bulky substituent at the *para*-position of multiple-substituent phenolic acids appears to be necessary for enhancing DPPH• scavenging activity, based on Figure 1a,b or Figure 2a,b. This conclusion is consistent with the study by Zhou *et al*. [14] indicating that the phenoxyl radical is initially developed at the 4-OH group by abstraction of the hydroxyl H atom, regardless of the molecular carbon skeleton. The additional possible resonance structures of multiple-substituted phenol acids would favor the stability of the resulting phenoxyl radicals [14]. Second, the presence of additional electron-donating and/or hydrophobic groups (e.g., OH and OCH_3_) in the *meta*-position for *para*-OH phenolic acid derivatives might enhance the radical scavenging activity (Figure 2b,e). The conclusion is consistent with previous studies [5,8,14]. Third, the presence of a hydrogen-bond donor group/electron-donating group on the *ortho*-position might enhance the radical scavenging activity of phenolic acids based on Figure 1b, Figure 2c, and Figure 2d. In addition, the bulky substituents and/or hydrogen-bond donors or hydrogen-bond acceptor groups at the X-position might enhance the activity of phenolic acids against free radicals based on Figure 1a, Figure 1b/ Figure 2b, Figure 2c and Figure 2d. The influence of side-chain groups on the activity is also important.

### 2.4. Application of QSAR Results of Phenolic Acid Derivatives to Previous Studies

The crucial structural components for the free radical scavenging activity concluded from our CoMFA and CoMSIA analyses were validated using phenolic acids from eight previous publications listed in Table 3according to the radical scavenging activity ranked from the highest to the lowest. The structure/activity relationship of phenolic acids from previous publications could be explained by structural criteria obtained from the present study.

The *para*-OH substituent important for the high free radical scavenging activity of phenolic acids was validated with previous research data (Table 3) [4,10,18]. The electron-donating and/or hydrophobic groups (e.g., OH and OCH_3_) in the *meta*-position-promoted activities of phenolic acids in the publications [4,10,14,19,20,21,22], satisfy the second criterion. For example, Abramovic *et al.* investigated the DPPH• radical scavenging activity of hydroxycinnamic acids that have in common a *para*-OH structur, but differ in their *meta*-substituents (*i.e.*, OH or OCH_3_) on the ring [19].

The hydroxyl group has electron-donating and hydrogen-bond donor properties, satisfying the second and third criteria, whereas the OCH_3_ group is hydrophobic, satisfying the secondary criterion. Therefore, the radical scavenging activity of phenolic acids was following such an order: caffeic acid (R_3_: OH) > sinapic acid (R_3_ and R_5_: OCH_3_) > ferulic acid (R_3_: OCH_3_) > umbellic acid (R_2_: OH) > *p*-coumaric acid (R_3_, R_5_, and R_2_: H). The presence of an *ortho*-OH on the ring acted as a hydrogen-bond donor group or electron-donating group enhancing the activities of phenolic acids in the publications [4,10,18,19,21], satisfying the third criterion. For example, umbellic acid (*ortho*-OH) showed greater activity than *p*-coumaric acid (*ortho*-H) [19], or 2,4-dihydroxybenzoic acid (*ortho*-OH) had a greater activity than *p*-hydroxybenzoic acid [4].

**Table 3:** Confirmation of functional structures and their DPPH• scavenging activities based on data from previous publications. 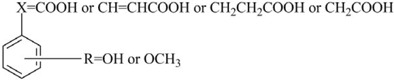

No.	Compounds	*para*-OH ^a^	*meta*-OH ^b^	*meta*-OCH_3_^c^	*ortho*-OH ^d^	X-	Bioactivity ^e^	Ref.
							**EC_50_^f^ (10^−5^ mol/L)**	[19]
1	caffeic acid *	+	+			CH=CHCOOH	2.6 ± 0.1	
2	sinapic acid	+		++		CH=CHCOOH	4.5 ± 0.2	
3	ferulic acid	+		+		CH=CHCOOH	4.9 ± 0.1	
4	umbellic acid *	+			+	CH=CHCOOH	8.6 ± 0.1	
5	*p*-coumaric acid	+				CH=CHCOOH	255 ± 64	
							**Inhibition %**	[18]
1	gallic acid	+	++			COOH	75 ± 2	
2	3,4-dihydroxyphenylacetic acid *	+	+			CH_2_COOH	70.8 ± 0.3	
3	2,3-dihydroxybenzoic acid *		+		+	COOH	46 ± 3	
4	protocatechuic acid	+	+			COOH	41.2 ± 0.6	
5	*α*-resorcylic acid *		++			COOH	0.60 ± 0.08	
6	*o*-hydroxybenzoic acid				+	COOH	0.11 ± 0.07	
7	β-resorcylic acid *	+			+	COOH	0.11 ± 0.07	
8	*m*-hydroxybenzoic acid *		+			COOH	0.07 ± 0.15	
							**IC_50_^f^ (μM)**	[20]
1	dihydrosinapic acid *	+		++		CH_2_CH_2_COOH	44.3	
2	dihydroferulic acid *	+		+		CH_2_CH_2_COOH	77.0	
3	sinapic acid	+		++		CH=CHCOOH	77.2	
4	ferulic acid	+		+		CH=CHCOOH	113.9	
5	vanillic acid *	+		+		COOH	250.0	
6	*p*-coumaric acid	+				CH=CHCOOH	2130	
							**TEAC ^g^ (mM)**	
1	gallic acid	+	++			COOH	3.92 ± 0.026	
2	syringic acid	+		++		COOH	1.33 ± 0.012	
3	protocatechuic acid	+	+			COOH	1.29 ± 0.007	[4]
4	2,4-dihydroxybenzoic acid *	+			+	COOH	1.27 ± 0.011	
5	*p*-hydroxybenzoic acid	+				COOH	0.059 ± 0.000	
6	*m*-hydroxybenzoic acid *		+			COOH	0.069 ± 0.000	
7	*o*-hydroxybenzoic acid *				+	COOH	0.052 ± 0.000	
8	benzoic acid *					COOH	0.006 ± 0.000	
							**Inhibition %**	[14]
1	syringic acid	+		++		COOH	90	
2	ferulic acid	+		+		CH=CHCOOH	60	
3	*p*-hydroxybenzoic acid	+				COOH	2	
							**EC_50_^f^ (10^−6^ M)**	[21]
1	gallic acid	+	++			COOH	5.1 ± 0.1	
2	2,5-dihydroxybenzoic acid *		+		+	COOH	7.6 ± 0.2	
3	caffeic acid *	+	+			CH=CHCOOH	12.1 ± 0.2	
4	syringic acid	+		++		COOH	12.3 ± 0.0	
5	ferulic acid	+		+		CH=CHCOOH	24.7 ± 0.4	
							**Inhibition %**	[10]
1	dihydrocaffeic acid *	+	+			CH_2_CH_2_COOH	93.9	
2	caffeic acid *	+	+			CH=CHCOOH	76.6	
3	sinapic acid	+		++		CH=CHCOOH	56.1	
4	ferulic acid	+		+		CH=CHCOOH	30.9	
5	*p*-coumaric acid	+				CH=CHCOOH	3.6	
6	*o*-coumaric acid *				+	CH=CHCOOH	3.5	
7	*m*-coumaric acid *		+			CH=CHCOOH	2.6	
8	*trans*-cinnamic acid					CH=CHCOOH	0.5	
							**EC_50_^f^ (μM)**	[22]
1	gallic acid	+	++			COOH	12.0	
2	protocatechuic acid	+	+			COOH	15.0	

^a^ the OH group on the *para*-position of the phenol ring; ^b^ the OH group on the *meta*-position of the phenol ring; ^c^ the OCH_3_ group on the *meta*-position of the phenol ring; ^d^ the OH group on the *ortho*-position of phenol ring; ^e^ the data of DPPH• scavenging activity according to cited references; ^f^ IC_50_ or EC_50_ is defined as the concentration of the compound to give a 50% of DPPH• scavenging activities; ^g^ The TEAC is defined as the concentration of Trolox (6-hydroxy-2, 5, 7, 8-tetramethylchroman-2-carboxylic acid) solution with equivalent antioxidant potential of a 1 mmol/L concentration of the compound; * the compounds are not in training set.

The DPPH• scavenging activity of phenolic acids in agreement with the steric property at the X-position was validated with previous research data too [18,20]. For example, 3,4-dihydroxy-phenylacetic acid with a bulky side chain (X: CH_2_COOH), showed greater activity than protocatechuic acid with a smaller side chain (X: COOH) [18]. Dihydroferulic (X: CH_2_CH_2_COOH) and ferulic acids (X: CH=CHCOOH) showed greater activities than vanillic acid (X: COOH) [20]. The phenolic acid with CH_2_CH_2_COOH at X-position had a greater activity than the one with CH=CHCOOH. This may be explained with the high capacity to donate protons for the “side-chain-saturated” phenolic acid [20].

## 3. Experimental

### 3.1. Experimental Design

Common food phenolic acids were randomly selected for the study. The 3D-QSAR models were established from the training data set of 17 phenolic acids. The experimental biological activity values were measures of the Trolox equivalent antioxidant capacity (TEAC) for DPPH• scavenging. For QSAR modeling, the DPPH• scavenging activity was converted into logarithmic activities. The techniques used to generate the QSAR models were comparative field analysis (CoMFA, SYBYL-X 1.2) and comparative molecular similarity index analysis (CoMSIA, SYBYL-X 1.2). Another seven phenolic acids were randomly chosen as the testing set to check the predictive powder of QSAR models. The critical structural characteristics of phenolic acids associated with free radical scavenging activities were analyzed. Literature data were also applied to verify the reliability of the structure-activity relationships. All chemicals, including phenolic acid standards, were purchased from Sigma-Aldrich (Shanghai, China).

### 3.2. DPPH Radical Scavenging Assay

The determination of DPPH• scavenging activities of the studied compounds were performed according to the previously reported procedure using a Synergy 2 Multi-Mode Microplate Reader (BioTek, Winooski, VT, USA) [23]. Briefly, each reaction mixture contained 100 μL of sample solutions and 100 μL of 0.2 mmol/L DPPH• solution. The TEAC is defined as the concentration of Trolox (6-hydroxy-2,5,7,8-tetramethylchroman-2-carboxylic acid) solution with equivalent antioxidant potential of a 1 mmol/L concentration of the compound. The DPPH• solution was added into each well to initiate the reactions, absorption at 515 nm was determined every minute for 40 min. The blank contained only 200 μL of solvent, and the control consisted of 100 μL of solvent and 100 μL of 0.2 mmol/L DPPH•. Triplicate tests were conducted. The DPPH• scavenging activity was expressed as pTEAC = −logTEAC.

### 3.3. Data Sets

The DPPH• radical scavenging assay was applied for studies of effect of structural properties of phenolic acids on free radical scavenging activity. A total of 17 phenolic acids (training set) were used to establish QSAR modeling (Table 2). Another seven compounds (test set) were applied to validate the final model.

### 3.4. Molecular Modeling and Alignment

**Figure 3:**
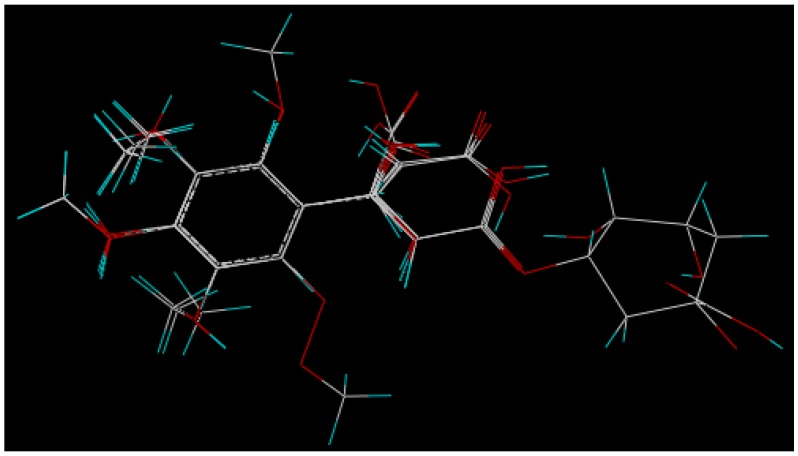
Alignment of the compounds used in the training set.

Molecular structure building was accomplished using the molecular modeling program from the SYBYL-X 1.2 software (Tripos, St. Louis MO, USA) on a Windows operation system. The energy minimizations of each structure were conducted with the Powell method using the Tripos force field [24], where a convergence criterion of 0.005 kcal/(mol Ǻ) was used as the termination of the Powell conjugate gradient algorithm and the maximum iterations were set to 1,000 steps. The partial atomic charges were calculated using the Gasteiger-Hückel method. Other parameters were default. Molecular superimposition of phenolic acids in the training set (Table 2) on the template structure was performed by database alignment method in SYBYL. The most active compound **11** was chosen as a template for superimposition and the common structure was the phenol, assuming that its conformation represented the most bioactive conformation of the phenolic acids. The 3D-view of 17 aligned molecules (training set) are shown in Figure 3.

### 3.5. Comparative Molecular Field Analysis (CoMFA) and Comparative Molecular Similarity Index Analysis (CoMSIA)

Comparative molecular field analysis (CoMFA) and comparative molecular similarity index analysis (CoMSIA) are the 3D-QSAR methods that use statistical correlation techniques to analyze the quantitative relationship between the biological activity for a set of compounds with a special alignment, and their three-dimensional electronic, steric properties, plus hydrogen bond donor/acceptor and hydrophobic properties specifically for CoMSIA. In this study CoMFA [25] was started with the QSAR option of SYBYL-X 1.2 in the Tripos force field. A 3D cubic lattice with a grid spacing of 2 Å in x, y, and z directions was created to encompass the aligned molecules in order to obtain the CoMFA and CoMSIA descriptor fields. The energies of steric (Lennard-Jones potential) and electronic (Coloumb potential) fields were calculated using a *sp*^3^ carbon atom as the steric probe atom and a + 1 charge for the electrostatic probe. The cutoff value for both steric and electrostatic interactions was set at 30.0 kcal/mol. In CoMSIA analysis [26], steric, electrostatic, hydrophobic, hydrogen bond donor and hydrogen bond acceptor properties were evaluated. Gaussian-type distance dependence was used to calculate the similarity indices. The default attenuation factor (α = 0.3) was used. There were no cutoff limits in CoMSIA analysis.

### 3.6. Partial Least Square (PLS) Analysis

The method of partial least square (PLS) implemented in the QSAR module of SYBYL was used to construct and validate the models. The CoMFA and CoMSIA descriptors were used as independent variables, and the biological activities in pTEAC values were used as dependent variables in PLS regression analysis to derive 3D-QSAR models using the standard implementation in the SYBYL-X 1.2 package [27]. The Leave-One-Out (LOO) was performed to obtain the optimum number of components, which consequently was used to develop the final non-cross-validated model determined by the cross-validation coefficient *q*^2^, the non-cross-validated coefficient *r*^2^ and its standard error s and F-test values for the model evaluation. To further assess the robustness and statistical confidence of the derived QSAR models, bootstrap analysis for 10 runs was performed. CoMFA and CoMSIA contour maps that intuitively reflect and analyze the different field effects on the activity [28] were obtained by interpolation of the pair-wise products between the PLS coefficients and the standard deviations of the corresponding CoMFA or CoMSIA descriptor values.

## 4. Conclusions

The structural criteria for free radical scavenging activity of phenolic acids have been deduced according to theoretical results of CoMFA and CoMSIA contour maps and serve to explain the real structure-activity relationships of selected phenolic acids from previous publications. Additionally, the structural criteria for free radical scavenging activity of phenolic acids could provide deeper insight into the mechanisms of their radical scavenging activities.
